# Modeling of Ethiopian Beef Meat Marbling Score Using Image Processing for Rapid Meat Grading

**DOI:** 10.3390/jimaging10060130

**Published:** 2024-05-28

**Authors:** Tariku Erena, Abera Belay, Demelash Hailu, Bezuayehu Gutema Asefa, Mulatu Geleta, Tesfaye Deme

**Affiliations:** 1 Department of Food Science and Applied Nutrition, Bioprocessing and Biotechnology Center of Excellence, Addis Ababa Science and Technology University, Addis Ababa P.O. Box 16417, Ethiopia; tariku.erena@aastu.edu.et (T.E.); demelash.hailu@aastu.edu.et (D.H.); 2 Food Science and Nutrition Research Directorate, Ethiopian Institute of Agricultural Research, Addis Ababa P.O. Box 64, Ethiopia; bezuayehug7@gmail.com; 3 Department of Plant Breeding, Swedish University of Agricultural Sciences, Sundsvägen 14, P.O. Box 101, SE 23053 Alnarp, Sweden; mulatu.geleta.dida@slu.se

**Keywords:** marbling, image processing, beef, rib-eye steak, beef texture, modeling

## Abstract

Meat characterized by a high marbling value is typically anticipated to display enhanced sensory attributes. This study aimed to predict the marbling scores of rib-eye, steaks sourced from the Longissimus dorsi muscle of different cattle types, namely Boran, Senga, and Sheko, by employing digital image processing and machine-learning algorithms. Marbling was analyzed using digital image processing coupled with an extreme gradient boosting (GBoost) machine learning algorithm. Meat texture was assessed using a universal texture analyzer. Sensory characteristics of beef were evaluated through quantitative descriptive analysis with a trained panel of twenty. Using selected image features from digital image processing, the marbling score was predicted with R^2^ (prediction) = 0.83. Boran cattle had the highest fat content in sirloin and chuck cuts (12.68% and 12.40%, respectively), followed by Senga (11.59% and 11.56%) and Sheko (11.40% and 11.17%). Tenderness scores for sirloin and chuck cuts differed among the three breeds: Boran (7.06 ± 2.75 and 3.81 ± 2.24, respectively), Senga (5.54 ± 1.90 and 5.25 ± 2.47), and Sheko (5.43 ± 2.76 and 6.33 ± 2.28 Nmm). Sheko and Senga had similar sensory attributes. Marbling scores were higher in Boran (4.28 ± 1.43 and 3.68 ± 1.21) and Senga (2.88 ± 0.69 and 2.83 ± 0.98) compared to Sheko (2.73 ± 1.28 and 2.90 ± 1.52). The study achieved a remarkable milestone in developing a digital tool for predicting marbling scores of Ethiopian beef breeds. Furthermore, the relationship between quality attributes and beef marbling score has been verified. After further validation, the output of this research can be utilized in the meat industry and quality control authorities.

## 1. Introduction

The marbling of meat refers to the distribution and amount of fat within the muscle fibers. It plays a crucial role in determining the tenderness, juiciness, and overall taste of beefsteaks. The level of marbling is closely linked to how well consumers perceive and accept the quality of the meat. From the moment of first classification to the point of consumer purchase, the meat business regularly uses visual inspection as a method of subjectively in analyzing and appraising various parts of the meat manufacturing process of beef quality [1]. The presence of intramuscular fat (IMF%), also known as marbling, is a critical factor that affects the eating experience of beef and lamb. It has a positive impact on the tenderness, flavor, juiciness, and overall enjoyment of the meat [2]. Marbling is recognized as a fundamental attribute of meat quality. It describes the amount and distribution of white fat spots that may be seen inside the longissimus dorsi (LD) cut’s lean muscle. It is thought to be one of the main elements that has a considerable impact on the overall quality of meat [3]. The degree of marbling is the primary evaluation criterion for assessing the quality of meat in the majority of developed countries, and there is frequently a strong relationship between marbling score and price [3].

The ribeye steak is a prized meat known for its marbling. It is often cooked quickly at high temperatures through methods like searing, pan roasting, or grilling. The American Meat Science Association’s 2015 findings [4,5] provide relevant information. The ribeye steak is obtained from the section between the 6th and 13th ribs and mainly comprises the Longissimus dorsi muscle. Taste and desirability can vary based on cattle breed and species. Marbling, assessed through objective textural analysis and subjective evaluations, is a key factor influencing beef’s sensory qualities and consumer choices. Marbling estimation is well established in beef industries worldwide, including the USA, Japan, Australia, and Korea. While sensory evaluation and instrumental analysis have been traditionally used for beef quality assessment, human panels may lack consistency and standardization. However, computer image analysis, pioneered by the Japanese meat grading association [6], is a reliable method for assessing marbling scores. Trustworthy statistical models derived from diverse human panels can facilitate prompt meat quality evaluation in the beef industry. Higher marbling levels significantly enhance tenderness, juiciness, flavor, and overall appeal of the steak, consistent with [5]. Marbling refers to the fat distributed within the muscle fiber of lean meat, excluding surface fat. In beef quality assessment, measures like small marbling number (SMN), small marbling area (SMA), and marbling ratio (MR) are critical. SMN quantifies the number of marbling flecks within the beef, providing an index for intramuscular fat which correlates with flavor and juiciness. SMA refers to the total area covered by marbling, indicating the potential for tenderness and taste. MR, the proportion of marbling to muscle, offers insights into the overall palatability and consumer satisfaction. These metrics contribute significantly to the grading system, where higher marbling equates to premium quality grades like Prime and Choice. The detailed analysis of these measures allows for a more understanding of beef quality beyond the traditional grading, catering to diverse consumer preferences and market demands. By leveraging these measures, producers can target specific quality attributes, enhancing the value and appeal of beef products in the marketplace. The small marbling number offers more details regarding the consistency and homogeneity of marbling, which can affect the overall tenderness and juiciness of the beef. It assists in evaluating the distribution of marbling throughout the muscle [7].

Breed affects intramuscular fat (IMF) quantity, which positively impacts tenderness [1]. Intramuscular fat enhances juiciness and imparts a rich, buttery flavor. Smith et al. (1982) found a positive correlation between marbling and beef flavor intensity and juiciness. Hyperspectral imaging can predict meat marbling by analyzing the reflectance or absorbance characteristics of meat samples. Different light wavelengths interact differently with intramuscular fat and muscle tissue, enabling identification and quantification of marbling levels [8]. The meat industry has created meat standards and grading systems with the goal of forecasting quality at various points in the beef supply chain in order to achieve this goal. Modeling represents a method that can be employed to achieve a better prediction of beef eating quality [9]. The degree of marbling is a significant measure of quality in numerous beef quality grading systems. It is an essential element that influences the flavor, juiciness, tenderness, and overall taste of meat. Furthermore, it is a significant aspect that consumers take into account when selecting meat products for purchase [10]. So far, visual appraisal remains the most commonly used method by the meat industry for the assessment of marbling degrees. Moreover, the determination of marbling degrees through chemical analysis has been a prevalent method. Nevertheless, both of these methods have drawbacks in that they are subject to bias and require a significant amount of time, or are time intensive. Furthermore, in order to surmount these challenges and achieve quick online grading of marbling degrees, multiple instrumental techniques (largely spectroscopic and imaging techniques) have been designed. However, up to now, in Ethiopian cattle meat, there have been no available data with respect to the methods and techniques employed for marbling analysis and the beef grading system. Therefore, the aim of the current research was to study the Ethiopian cattle meat marbling score from three different cattle types (four cattle types from each cattle, totaling twelve). In addition, this study developed a model for rapid determination of the Ethiopian beef marbling score using data obtained from sensory analysis and digital image processing for better prediction of meat quality.

## 2. Materials and Methods

### 2.1. Meat Samples

This research encompassed twelve cattle from three unique Ethiopian breeds, aged between 18 and 24 months. The majority hailed from the Borana zone, while the Sheko and Senga breeds were procured from the southwestern regions and Akobo district of Gambella, respectively. For analysis, sirloin and chuck cuts were selected to represent the Boran, Senga, and Sheko types. These cattle were then moved to specific areas within slaughterhouses for a 12-h fast, with access to water until their processing. The next day, they were processed, and the carcasses were immediately placed in a 2 °C refrigeration unit. The following day, carcass partitioning occurred between the sixth and thirteenth ribs on the left side. Trained graders then meticulously assessed and assigned quality grade (QG) and yield grade (YG) values to each carcass [5].

Following the grading process, the carcasses were transported to a cutting room for additional processing. The carcasses were deboned and segmented into three primary cuts: loin, chuck roll, and ribeye. The cuts from the left side of the carcasses were meticulously wrapped and subjected to a 14-day aging process in a refrigerator maintained at a temperature of 2 °C. After the aging period, the cuts were further subdivided into smaller blocks for subsequent chemical and sensory evaluation [11]. Before analysis, all muscle samples were removed of fat and epimysium. The steak meat blocks were then sliced perpendicular to the orientation of the muscle fibers. The sliced samples were subsequently frozen at a temperature of −18 °C [12].

### 2.2. Reference Analysis

#### 2.2.1. Determination of Fat Content

Using a Soxhlet system, petroleum ether was employed to extract the crude fat content. In this analysis, a 2-g subsample from the meat sample was utilized for fat extraction. The sample underwent continuous extraction with petroleum ether for 5 h. Following extraction, the sample was removed from the extractor and allowed to dry in an oven for 2 h (AOAC, 2000). Subsequently, the sample was cooled and weighed to determine the optimal concentration of ether extract [13].

#### 2.2.2. Texture Profile Analysis

Meat samples prepared in a cylindrical shape from the three beef breeds were subjected to texture profile analysis (TPA). Texture profile analysis (TPA) was conducted using a Lloyd Instruments AMETEK™ TA1 universal texture analyzer equipped with a 500 N load cell and a cylindrical probe with a diameter of 20 mm. The instrument was configured to perform the test in the compression direction with a preload of 0.1 N and a 3 mm/s preload speed. The extension rate during testing was maintained at 1 mm/s, and the test limit was set at a compression distance of 75% of the height of the meat sample [14]. The tenderness (N), toughness (N), cohesiveness (N), and consistency (Nmm) were measured. All the measurements and TPA data were acquired using NEXYGEN Plus data acquisition and analysis software (01/5050 NexygenPlus 4.1, AMETEK™). 

#### 2.2.3. Sensory Analysis of Beef Marbling Score

Marbling score is a critical quality attribute, as it is directly correlated with the sensory attributes of tenderness and juiciness that are highly valued in meat products. The sensory evaluation was conducted in accordance with the established protocol within the sensory analysis laboratory of the Department of Food Science and Nutrition at Addis Ababa Science and Technology University. The assessment panel comprised twenty trained individuals, all of whom were accurately selected from the department’s faculty, ensuring a high level of expertise and familiarity with the sensory characteristics of the cuts, and these subjects were selected using triangle test methods. Then, samples were presented randomly under fluorescent lighting to characterize the color of the muscle and intramuscular fat content. The panelists were instructed to score the presence of marbling on a scale ranging from 1 (devoid) to 7 (very abundant), which was used to determine the marbling score of the meat sample [15].

### 2.3. Image Analysis

#### 2.3.1. Image Acquisition

Image acquisition was conducted on meat samples prepared as per the description in Section 2.1. From each breed, meat samples were obtained from four different species. A similar procedure was replicated in animals of the same group eight times. The total number of meat samples used for the imaging was thus 3 × 4 × 8 = 96. A duplicate image was captured from each of the two sides of every meat sample presented to the camera. Consequently, a total of four images were captured for each individual meat sample. This process resulted in the collection of 384 images from the 96 meat samples prepared for image analysis. High-resolution images of the meat specimens were captured using a Canon EOS 6D Mark II digital camera boasting a 24-megapixel resolution capacity. The imaging process was carried out under controlled illumination to guarantee consistent and accurate results. The camera utilized for this purpose was manufactured by Canon (EOS 6D Mark II digital camera, Toyko, Japan).

Images of the meat samples were collected under controlled illumination using a Canon EOS 6D Mark II digital camera with a resolution of 24 mega-pixels. The samples were positioned within a uniformly illuminated image acquisition slot crafted from a gray magnesium board in a box shape. The camera was mounted at the top of the chamber, focusing downward toward the samples through a circular hole. The entire image acquisition process was systematically controlled from a laptop (Lenovo, Core I7, Beijing, China) using camera software (EOS Utility 3, Canon, Toyko, Japan). Further specifications on the image acquisition chamber and illumination setup can be found in [16].

#### 2.3.2. Image Processing and Feature Extraction

**Figure 1:**
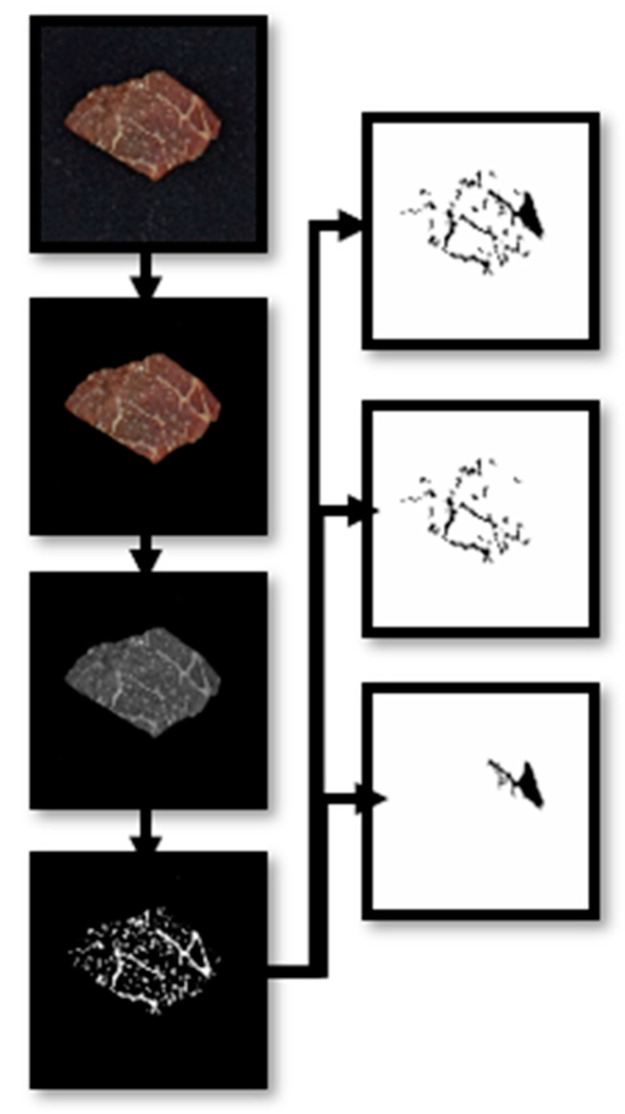
Summarized illustration of the image processing procedure.

Figure 1 shows a simple depiction of the image processing process. The ImageJ program (National Institutes of Health, New York, NY, USA, Version 1.8.0) was used for image analysis and feature extraction utilizing a batch image processor tool to execute various image analysis procedures. The overall image analysis process included resizing the original images, establishing a measurement scale, binarizing, and conducting qualitative and quantitative analyses of marbling flecks.

The original image of the meat samples with a wide background was resized to a 500- by 500-pixel size. The color shareholding of the resized image was then performed to remove the background from the region of interest. Using an automatic color shareholding tool, the color model was set to Lab* with L = 0 by 255, a = 0 by 255, and b = 0 by 129 threshold levels. The resulting image was then subtracted from the original image to yield a new grayscale image, which was subsequently converted to a binary image using the make binary function in the software. Depending on the marbling fleck size, the binary image was further processed to generate three images, which represent the following: (1) all marbling flecks, (2) small marbling flecks, i.e., flecks between 1 mm^2^ and 50 mm^2^; and (3) large marbling flecks, i.e., flecks > 50 mm^2^.

The image features used to characterize the marbling flecks were the small marbling fleck number (SMN), the area of small marbling flecks (SMA), the overall number of marbling flecks (TMN), the total area of marbling flecks (TMA), and the marbling ratio (MR), calculated by dividing TMA by the area occupied by the beef. Finally, the fineness index (F) was calculated by dividing the small marbling fleck number (SMN) by the beef area [6]. Image processing was used to extract important information and variables for the prediction of marbling scores. These key indicator variables (small marbling number, small marbling area, marbling ratio) for marbling score were identified and applied in image-based marbling score predictions in some other countries [1,17]. Therefore, since we implemented established procedures for image processing, we believe potential errors that affect the accuracy of the extracted information were avoided. While developing the prediction model, we implemented the necessary procedures to ensure potential sources of error were mitigated. Starting with sample representativeness, we took sufficient precautions to avoid misleading results by carefully treating replicate samples. Finally, the developed prediction model has been tested on test data, which were unseen during the training phase. Therefore, the developed prediction model followed a standard procedure and can be used as a reliable model for predicting marbling scores.

### 2.4. Development of the Marbling Score Estimation Model

The data generated from the beef image features were systematically grouped into training and test sets in a ratio of 80:20. Care was exercised during the consolidation of duplicate data, with each row from a duplicate image systematically assigned to either the training set or the test set group. The development of the marbling score estimation model involved the utilization of the extreme gradient boosting (XGB) regression algorithm. A menu-driven program, Solo (version 8.9.1; Eigenvector Research, Inc., Manson, WA, USA), was used to create the regression model. An XGB-based approach was employed to construct the regression model. This method enhances the accuracy of regression predictions by combining multiple decision trees into an ensemble [17]. This method permits us to examine variable importance weights and is recognized to show exceptional performance in solving regression problems [18]. The extreme gradient boosting (XGBoost) machine learning algorithm was used to develop a marbling score prediction model. The method was selected because it performed better than other machine learning algorithms such as SVM, PCR, ANN, and PLS in a preliminary trial. Furthermore, XGBoost has demonstrated superior or comparable prediction performance compared to other machine learning algorithms such as SVM [19].

### 2.5. Statistical Analysis

The means of the beef meats that were put to the test were compared using ANOVA testing as well as Tukey’s post hoc test. To find out if there was a significant difference level (LSD), *p* < 0.05 was used. Principle component analysis (PCA) was used to study correlations among various quality descriptors and the cattle breeds.

## 3. Results and Discussion

### 3.1. Reference Analysis

Fat content, instrumental texture analysis, and sensory marbling score determination were conducted. The means ± standard deviations of the results across the different cattle types are summarized in Table 1.

#### 3.1.1. Fat Content

The means and standard deviations of the fat measurements derived from the replicate data are shown in Table 1. The results displayed in Table 1 demonstrate the fat content of the meat cuts derived from the three cattle breeds: Boran, Senga, and Sheko. The Boran cattle cuts (sirloin and chuck) had relatively high amounts of fat (12.68 ± 0.59 and 12.40 ± 0.63%, respectively). These results are in agreement with those of [20], who reported a fat content of 2.6 ± 4.7 in a retail-ready meat sample. A low percentage of fat content was observed in the cuts from the Sheko breed (11.40 ± 0.87 and 11.17 ± 1.03) sirloin and chunk, respectively. This was in line with the study conducted by Robert [21], who found the beef meat total fat to be 10.72 ± 0.10%. However, the percentage in the present study was greater than that in the study conducted by Timketa Dagne et al. [22], in which the fat percentage in Boran was 5.82 ± 0.58%. The difference might be due to the low fat content of the meat, which is because the fat content of beef depends on the breed, age, and diet of the animal and because the use of growth hormones in cattle can increase the fat content of the meat [23]. Moreover, in the study of Hongbin et al. [24], the fat percentage in lamb muscle (2.42 ± 1.33) was lower than that in the present study, in which the fat contents of the three breeds, Boran, Senga, and Sheko, were 12.68 ± 0.59 and 12.40 ± 0.63, 11.59 ± 0.70 and 11.56 ± 0.47 and 11.40 ± 0.87 and 11.17 ± 1.03, respectively, for both sirloin and chuck. Furthermore, the results of the present study were greater than those of Lijalem et al. [25], who reported that the fat percentage of beef was 5.4 ± 0.8. In addition, a study by Williams [26] showed that the fat content of beef was in agreement with that of the current study, with Senga and Sheko cattle types (11%). The current study showed that fat percentage is an important factor that affects the tenderness of meat, as shown in Table 1. Fat percentage and tenderness were greater in Boran (Boran sirloin contained 12.68 ± 0.59 and 7.06 ± 2.75 fat and tenderness, respectively). Similarly, studies have shown that higher fat content can lead to improved meat tenderness. Calkins and the authors of [27] reported that increasing the fat content of beef from 10% to 20% improved its tenderness. Several studies have indicated that there is a positive correlation between meat tenderness and marbling. The findings of the present study demonstrated that the tenderness and marbling scores of Boran sirloin cattle (7.06 ± 2.75 and 4.28 ± 1.43, respectively) were significantly greater than those of the Sheko and Senga cattle breeds. This was consistent with the findings of Muelaet [28], who demonstrated that marbling was strongly related to meat tenderness in beef from different breeds [29], suggesting a positive relationship between marbling and meat tenderness in beef.

#### 3.1.2. Muscle Tenderness

Tenderness is widely recognized as the primary attribute of eating quality that strongly impacts consumer pleasure with meat products, particularly in the case of beef [17]. The softness or tenderness of the meat slices/cuts from the three cattle types chosen for the present study, Boran, Senga, and Sheko, were 7.06 ± 2.75 and 3.81 ± 2.24; 5.54 ± 1.90 and 5.25 ± 2.47, and 5.43 ± 2.76 and 6.33 ± 2.28, respectively. The results of that study were lower than those of [30], in which the overall tenderness of beef chucks was 7.0%. In contrast, the findings of the present study were greater than the results reported by [31], who reported WBSF values of 4.06, 4.73, 4.17, 4.43, and 3.86 for the Angus, Bonsmara, Brahman, Charolais, and Nguni cattle breeds, respectively. Similarly, Ref. [32] reported that the mean beef tenderness was 14.26 ± 3.9, which was greater than that in the present study. The difference in tenderness might not be due to the breed effect. According to [33], breed effects did not have an impact on meat tenderness, as evidenced by the fact that the Warner–Barrler shear force (WBSF) and sarcomere length (SL) were not affected by breed. Nevertheless, approximately 45 min after slaughter, noticeable alterations in the structure of the myofibrils occurred. According to the findings of [34], the breed of cattle affects the average pH, tenderness (as determined by shear force), cooking loss, water holding capacity, water content, protein content, and fat content of the longissimus dorsi muscle. After a 24-h period, changes in the properties of the muscle fiber bundles were observed. Although meat tenderness is recognized to be directly related to the myofibrillar structure, these results suggest that meat tenderness may be enhanced by muscle fiber bundle characteristics at 24 h post slaughter [32].

#### 3.1.3. Marbling Score

Marbling is known to affect the flavor of beef and is closely linked to consumer preference for beef steaks; hence, it plays a vital role in many beef quality grading systems [35]. It is essential to precisely measure and report marbling levels to cattle producers to be acknowledged and encouraged within production systems or to be included as a grading criterion for carcasses. Furthermore, for correct recognition and value evaluation of beef, the marbling level must be included in cattle categorization and payment systems [35]. The present study showed that the mean marbling scores of sirloin and chunk of Boran, Senga, and Sheko cattle were 4.28 ± 1.43, 3.68 ± 1.21 and 2.88 ± 0.69; 2.83 ± 0.98 and 2.73 ± 1.28; and 2.90 ± 1.52, respectively (Table 1). In the present study, Boran cattle had higher marbling scores than Senga and Sheko cattle, and the sirloin cut was observed to have the highest marbling score. The authors of [36] reported that the mean marbling score of Hanwoo beef in Korea was 6.7, which was higher than that in the present study. The Boran cattle type had the highest marbling score, and the Sheko cattle type had the lowest marbling score. Similarly, the findings of the present study showed that the mean marbling scores of Boran, Senga, and Sheko beef (4.28 ± 1.43, 3.68 ± 1.21 and 2.88 ± 0.69; 2.83 ± 0.98 and 2.73 ± 1.28; 2.90 ± 1.52, respectively; Table 1) were significantly lower than those of Wagyu and Hanwoo beef [37], who reported a mean marbling score of 6.9 for Hanwoo beef, with significant variations observed between different cuts of meat. In addition, a significant difference was observed only between sirloin cuts of Boran and Sheko (Boran sirloin: 4.28 ± 1.43 and Sheko sirloin: 2.73 ± 1.28) cattle meat cuts. This finding was in line with the results of [38], who showed significant differences in marbling scores between different breeds of beef cattle, with Japanese Wagyu cattle having the highest marbling scores.

### 3.2. Relationships among Marbling Score, Fat Content, Instrumental Texture Profiles and Cattle Types

**Figure 2:**
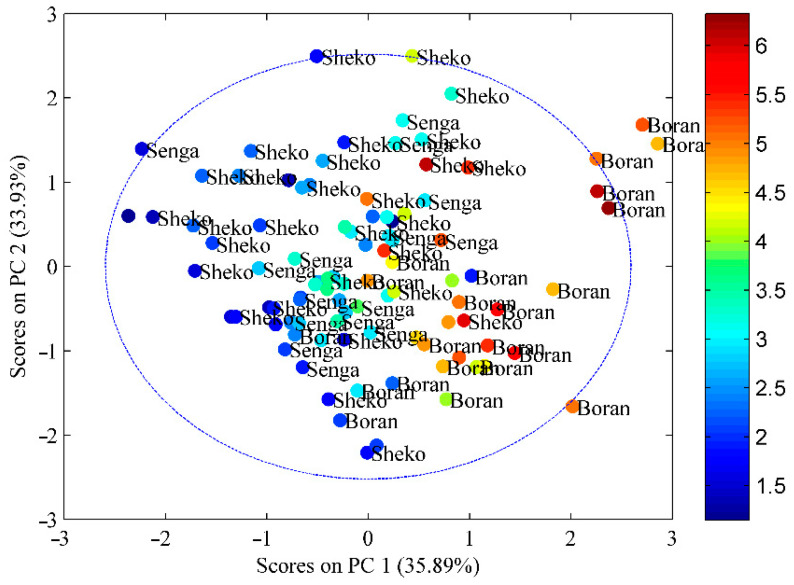
Marbling score in three Ethiopian cattle types.

The principal component plot in Figure 2 shows the correlations between various quality descriptors and the cattle breeds, namely, Boran, Senga, and Sheko. The plot revealed that key quality indicators, such as marbling level and fat content, are situated near the upper left quadrant adjacent to Boran cattle. The illustration particularly recognized the sensory quality of Boran meat cuts from the remaining two cattle. The unique sensory attribute of Boran is also indicative of its overall acceptability, as it was also demonstrated with the highest intramuscular fat content (12.40 to 12.68%).

The relative closeness of Senga to Boran is also indicative of similarities in some sensory attributes compared to the remaining Sheko meat cuts. This is also further supported by the fat content results presented in Table 1. In addition to the intramuscular fat (IMF) content, the average marbling score of Senga meat cuts is relatively close to that of meat cuts from the Boran group. Despite the relative closeness of the Senga samples to Boran, the Senga and Sheko samples are closer to each other than the Boran samples. This is also reflected in the tenderness, marbling score, and IMF content of the samples, as illustrated in Table 1. These variations could be due to different reasons. An investigation by [39] revealed that the similarity or closeness of meat quality among different cattle breeds can be ascribed to a range of factors, including genetic elements, muscle composition, and the distribution of fat. These factors can potentially impact the tenderness, marbling, flavor, and overall eating experience of meat. Similarly, genetic factors play a significant role in the variations observed in meat quality traits among different cattle breeds. Each breed possesses a unique genetic background that influences these traits. Cattle breeds that have undergone selective breeding to enhance desirable meat characteristics, such as marbling or tenderness, tend to exhibit similar meat quality. Conversely, breeds that have not been specifically bred for meat quality are more likely to display greater variability in their meat characteristics. In addition, the ratio of fast-twitch to slow-twitch fibers, for example, might affect the tenderness and other qualities of meat by affecting muscle composition. According to [40], some breeds may have a greater proportion of particular fiber types, which results in more consistent meat quality within those breeds. Additionally, the amount, type, and distribution of intramuscular fat (marbling) have a significant impact on the tenderness, flavor, and juiciness of meat. Breeds with higher levels of natural marbling tend to provide meat of more consistent quality within the breed [41].

**Figure 3:**
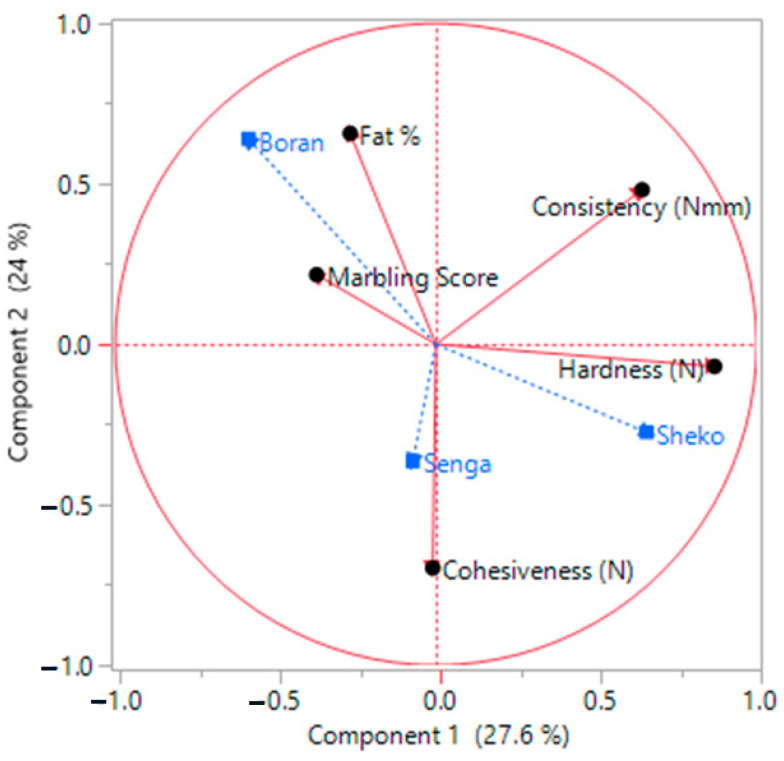
Overview of variation among marbling score, fat content, and texture profiles within the three cattle types.

The Senga and Sheko samples found in the lower quadrant are positioned far from the Boran samples, indicating a low marbling score and fat content (Figure 3). In contrast to those of Boran, the two samples, particularly Sheko, are adjacent to the hardness, indicating that the meat toughness was measured by an instrumental texture analyzer. Studies have indicated that marbling is a function of collagen content, collagen solubility, the effectiveness of intramuscular connective tissue, the diameter of fibers, the absence of shortening in sarcomeres, and disorganization of the perimysia, all of which result in better tenderness [5]. In the present study, variations were observed among the three cattle types (Boran, Senga, and Sheko) in their cuts. Several factors can influence the features of meat cuts, including the breed of cattle, age, diet, and management practices. These variables can give rise to differences in the meat quality, texture, and flavor of meat cuts across different breeds of cattle [42].

The marbling score, fat concentration, and texture profile are significant factors that contribute to the overall quality of meat products. Nevertheless, significant variations exist among these factors, exerting an impact on the flavor, tenderness, and overall palatability of the meat. The marbling score, commonly used as a quality metric for beef products, reflects the quantity and distribution of intramuscular fat within a piece of meat. Higher marbling scores are generally associated with improved flavor and tenderness, making them desirable for both consumers and producers. However, there can be significant variation in marbling scores between different cuts of meat, as well as among different animals or breeds. Similarly, as shown in Figure 2, the third quadrant marbling score and fat percentage were greater in the cuts of Boran cattle and Sheko cattle, which were represented by muscle hardness.

Similarly, cohesiveness was predominantly observed in the Senga cuts. Wagyu beef is renowned for its high marbling score, which contributes to its rich flavor and buttery texture [9]. In contrast, leaner cuts such as sirloin or round cuts may exhibit lower marbling scores, resulting in a tougher, less flavorful product. Marbling, which describes the intramuscular fat deposits found in the muscle tissue of cattle, is frequently cited as a crucial component in defining the flavor and quality of beef. It is vital to remember that marbling has some restrictions, even though it is frequently cited as a sign of beef quality. Thus, while marbling score is one established measure of cattle quality, there are certain limitations, such as subjectivity, genetics, and customer preferences. New findings in data analytics and machine learning methodologies, by way of prediction model improvement, enable the creation of prediction models that are more reliable and accurate [42]. 

Fat content is an additional significant factor that can influence the flavor and texture of meat products. A higher fat content is generally associated with improved flavor and tenderness, as well as increased juiciness [9]. However, there can be significant variation in fat content between different cuts of meat, as well as between different animals or breeds. As an illustration, beef from grass-fed cattle is typically leaner than beef from grain-fed cattle. This difference in fat content can contribute to a less flavorful and less tender meat product [43].

Texture profiles are yet another important factor that can influence the acceptability of meat products. Texture can be affected by various factors, such as marbling score, fat content, cooking method, and aging [9]. In general, tender meat with a fine texture is considered more desirable than tough, coarser meat. However, there can be significant variation in texture profiles even within a single cut of meat, which can make it difficult to consistently produce high-quality products.

The overall quality of meat products is influenced by several crucial factors, including marbling score, fat content, and texture profile. Nevertheless, there can be notable variations in these aspects across different cuts of meat, as well as among various animals or breeds. Understanding this variation is important for both consumers and producers, as it can help to ensure that high-quality products are consistently produced and marketed [44].

The most important parameter for texture profile analysis (TPA) was hardness, which has a significant impact on initial tenderness [45]. The present study findings, shown in Table 2 (Senga sirloin and chuck, 5.52 ± 1.58 and 5.98 ± 1.92 and Sheko chuck: 5.68 ± 1.70) were in line with the study by Caine (the hardness of the beef rib steaks was 6.04 ± 0.99). The cohesiveness parameter of the texture profile analysis (TPA) did not reach the required significance level of 0.15, preventing its inclusion in the stepwise regression models used to analyze the sensory characteristics associated with textural properties [44]. Similarly, the present study showed that the tenderness of the Sheko chunk (6.33 ± 2.28) was similar to that reported in the study by [46] (5.9 ± 0.6). In addition, according to the results of the present study, the tenderness of the three cattle types on two different cuts (Senga: sirloin: 5.54 ± 1.90, chunk: 5.25 ± 2.47; Sheko: sirloin: 5.43 ± 2.76, chunk: 6.33 ± 2.28) was in agreement with the findings of [47], who showed that the muscles of Holstein Friesian bulls were 6.06 ± 0.199, 5.56 ± 0.199, 5.65 ± 0.199 and 5.97 ± 0.199, respectively. Another study reported [32] that beef muscle tenderness was 26.77 ± 2.202, which was not in line with the present study.

According to previous findings, surface visual traits (marbling) are significantly influenced by breeding methods [48]. The examined breeds, Boran, Senga, and Sheko, have very little marbling (sirloin: 4.56 ± 1.60, chuck: 3.82 ± 1.06, sirloin: 3.03 ± 0.68, chuck: 2.73 ± 0.90, and sirloin: 2.88 ± 1.42, chuck: 2.92 ± 1.67), and there were no differences in marbling means (*p* < 0.01) between the various cattle types. These findings are consistent with those of [32], who showed that the marbling scores of the beef breeds Angus, Bonsmara, Brahman, Charolais, and Nguni were 2.06,1.91, 3.79, 2.18, and 2.28, respectively. The present study showed that the greater the marbling score of the meat cuts, the greater the tenderness of the meat. Likewise, Ref. [32] reported that the amount of marbling has an important effect on meat tenderness. In the present study, Sheko cattle types had relatively low marbling scores, possibly due to differences in cattle type, diet, and geographical location [49].

### 3.3. Principal Component Analysis

As shown in Figure 2, principal component analysis (PCA) was used to reduce the number of variables and acquire greater knowledge of their interactions. The first and second main components (PCs; PC1 and PC2, respectively) together accounted for 51.6% of the total variation. Figure 2 illustrates the strong correlation between marbling score and fat content, revealing a positive association between these two variables. In the region where PC1 had positive loading, cohesiveness and hardness were dispersed. Furthermore, cohesiveness and hardness were dispersed in the region with positive loading on the component, while uniformity was located in the region with positive loading on component 1. Cohesiveness and hardness were spread in the region with positive loading on the component, but uniformity was located in the region with positive loading on component 1.

### 3.4. Prediction of the Marbling Score Using Image Features

**Figure 4:**
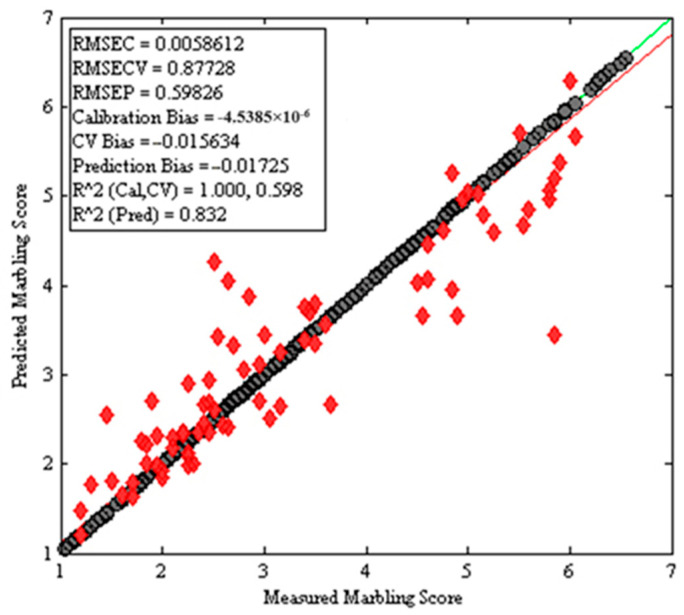
XGB regression plot using image features as a predictor of marbling score.

**Figure 5:**
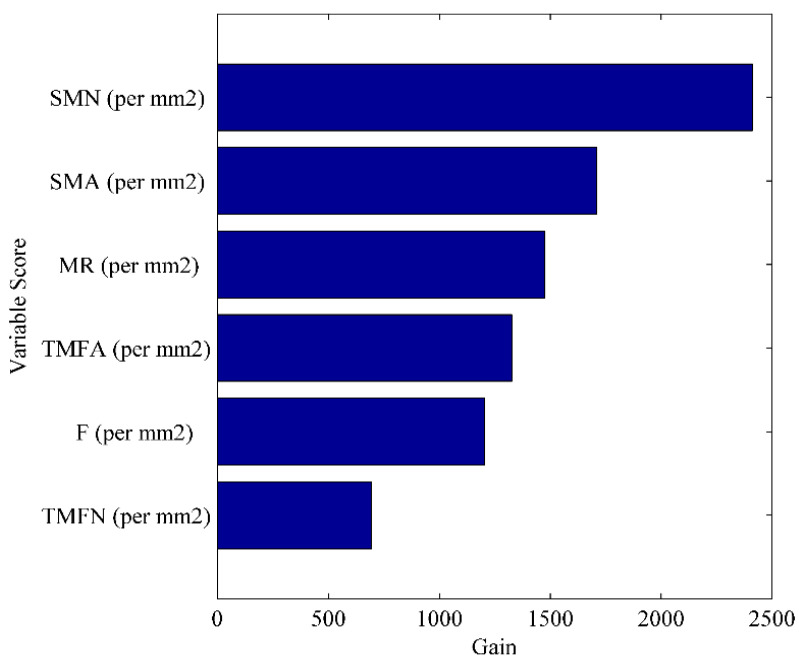
Variable importance plot indicating contributions of image features for the obtained prediction performance.

A regression model was developed using XGB after preliminary findings from many machine learning techniques, such as partial least squares (PLS), principal component regression (PCR), support vector machines (SVM), and artificial neural network (ANN) methods, were compared. Overall, six predictor variables, which were obtained from the attributes of the processed images, were used to create the XGB regression model. A Venetian blinds cross validation strategy with 10 window sizes was implemented during the model development, with R^2^ (pred.) of 0.83 and an RMSEP of 0.60, as shown in Figure 4. With the exhibited prediction performance, the model can be applied for rapid estimation of marbling scores using images acquired using digital cameras. The prediction model was built using the XGBoost machine learning algorithm. One of the key advantages of this model is that it allows for interpretability because of the variable importance plot. Figure 5 in the manuscript illustrates the top important variables for the obtained prediction result. A detailed interpretation of the VIP plot has been given in lines 452 to 483. However, it is not straightforward to absolutely rely on the VIP plot for the model’s interpretability.

Further investigation of the importance of the variables in the prediction model was carried out using a VIP plot (Figure 4). The small marbling number (SMN), small marbling area (SMA), and marbling ratio (MR) were the most important variables affecting the developed regression model.

The marbling score prediction model developed in this study using machine learning algorithms uses a combination of color and texture features extracted from digital images of beef cuts to predict marbling scores [50].

Marbling is a critical element when measuring the quality or value of beef, as it has a significant effect on the flavor, tenderness, and juiciness of the meat. The quantity and distribution of marbling in beef cuts can significantly impact these key sensory characteristics. To quantify marbling in beef, numerous measures were used, including a small marbling number, small marbling area, marbling ratio, total marbling fleck area, and total marbling fleck number. These measurements aid in establishing overall meat quality and providing an exhaustive assessment of marbling in cattle.

The quantity of marbling flecks in beef cuts that are smaller than 0.5 mm in diameter was quantified by the small marbling number (SMN). Higher SMN readings indicate greater meat quality, which is a widely used indication of cattle quality. The total marbling fleck number (TMFN) per mm^2^ in the present investigation, however, was found to be the least effective imaging feature in terms of prediction ability. According to Figure 5 from the current investigation, a value of 750 for this metric corresponded to the fewest image features for the expected performance. This finding suggests that the total marbling fleck number (TMFN) per mm^2^ may be a useful measure for predicting the quality of beef cuts [51]. Likewise, the results of the current study revealed that the highest image features for the obtained prediction performance were observed at values above 2350 per mm^2^ for the small marbling number (SMN) (Figure 5). These findings are in line with previous research by [6], who demonstrated a significant association between SMN and beef tenderness, indicating that this measure could be a valuable predictor of meat quality. Collectively, these results suggest that both the SMN and the TMFN per mm^2^ may be useful measures for predicting the quality of beef cuts.

The small marbling area (SMA) is a measure that calculates the total area of marbling flecks that are less than 0.5 mm in diameter in beef cuts. The present study showed that both the small marbling number (SMN) and small marbling area (SMA) had higher values than did the other image features for predicting the quality of beef cuts. These findings were in agreement with previous research by [52], which showed that higher SMA values, along with SMN values, are generally associated with better quality beef. Additionally, Cho et al. reported positive relationships between SMA and beef flavor, tenderness, and overall acceptability. Collectively, these results suggest that the SMA, along with the SMN, may be useful measures for assessing the quality of beef cuts.

The marbling ratio (MR) is a measure of the quantity of intramuscular fat in a beef cut. This measure is often used as a grading system for beef, with higher marbling ratios indicating better quality meat. A study by [53] discovered that the marbling ratio has a substantial impact on the flavor, softness, and general acceptance of beef.

The total marbling fleck area (TMFA) is a measure of the total area of marbling flecks in a beef cut, irrespective of size. This measure was often used in combination with other measures of marbling to provide a more comprehensive picture of beef quality. A study by [54] showed that TMFA was positively correlated with beef flavor, tenderness, and overall acceptability.

The total marbling fleck number (TMFN) is a measure of the total number of marbling flecks in a beef cut, regardless of size. This measure is another way to assess the amount of marbling in a beef cut and is often used in conjunction with other measures of marbling. A study by [35] reported that the TMFN was significantly correlated with beef tenderness and overall acceptability. Overall, while measures of marbling traits provide useful methods for assessing the quality of beef cuts, it is important to remember that several other factors influence beef quality as well. Factors such as the breed of cattle, their feeding practices, and the methods used for processing can all significantly influence the quality of beef. Therefore, these measures of marbling traits are recommended for use in conjunction with other assessment tools to obtain a more comprehensive evaluation of beef quality. This approach can help producers and consumers make informed decisions about the quality and value of beef cuts. The developed marbling score prediction model could be valuable in the meat industry because it provides rapid assessment of meat quality. These marbling score prediction models have the potential to improve the efficiency and accuracy of grading beef meat, which can benefit both producers and consumers. However, further research is needed to validate and refine these models, as well as to explore their applicability to different beef cuts and breeds.

## 4. Conclusions

The marbling score stands as a pivotal metric in the meat industry, particularly for Ethiopian cattle meat, where it serves as an indicator of quality. The integration of image and sensory data analysis into the evaluation process not only enhances the accuracy of marbling assessments but also facilitates the development of predictive models tailored to the unique characteristics of Ethiopian beef. These models are instrumental in providing a rapid and reliable estimation of marbling scores, thereby streamlining the quality control process. The adoption of model-based predictions is highly advantageous, offering a multitude of benefits, including improved consistency in meat quality, optimized consumer satisfaction, and bolstered economic value of the meat products. The implementation of these models is recommended for the advancement of the Ethiopian meat industry, ensuring that it remains competitive and maintains high standards. Ongoing research and development are essential to adapting the model to evolving industry standards and consumer preferences. The ultimate goal is to ensure that the marbling score remains a robust and reliable indicator of quality, contributing to the reputation and success of Ethiopian cattle meat in the global market. This will not only bolster consumer confidence but also support sustainable industry practices.

## Figures and Tables

**Table 1:** Fat content of (raw) beef, tenderness, and marbling score from three cattle, namely, Boran, Senga, and Sheko (% on dry weight basis).

Cattle	Beef Cuts	Fat %	Tenderness (N)	Marbling Score
Boran	Sirlion	12.68 ± 0.59 ^a^	7.06 ± 2.75 ^c^	4.28 ± 1.43 ^f^
	Chank	12.40 ± 0.63 ^a^	3.81 ± 2.24 ^d^	3.68 ± 1.21 ^g^
Senga	Sirlion	11.59 ± 0.70 ^ab^	5.54 ± 1.90 ^e^	2.88 ± 0.69 ^gh^
	Chank	11.56 ± 0.47 ^ab^	5.25 ± 2.47 ^e^	2.83 ± 0.98 ^gh^
Sheko	Sirlion	11.40 ± 0.87 ^b^	5.43 ± 2.76 ^e^	2.73 ± 1.28 ^h^
	Chank	11.17 ± 1.03 ^a^	6.33 ± 2.28 ^c^	2.90 ± 1.52 ^h^

Means followed by different superscripts in the same column are significantly different (*p* < 0.05).

**Table 2:** Toughness, cohesiveness, and tenderness and marbling score characteristics of the sirloin and chuck cuts of the selected cattle types of Ethiopia.

Cattle Types	Muscle Location	Hardness (N) Mean ± Stadv	Cohesiveness (N) Mean ± Stadv	Tenderness (Nmm) Mean ± Stadv	Marbling Score Mean ± Stadv
Boran	Sirloin	11.64 ± 5.02 ^a^	−0.03 ± 0.02 ^e^	7.06 ± 2.75 ^f^	4.56 ± 1.60 ^dh^
Boran	Chunck	3.55 ± 2.15 ^b^	−0.03 ± 0.03 ^e^	3.81 ± 2.24 ^bc^	3.82 ± 1.06 ^h^
Senga	Sirloin	5.52 ± 1.58 ^c^	−0.05 ± 0.02 ^e^	5.54 ± 1.90 ^cd^	3.03 ± 0.68 ^h^
Senga	Chunck	5.98 ± 1.92 ^c^	−0.04 ± 0.01 ^e^	5.25 ± 2.47 ^cd^	2.73 ± 0.90 ^h^
Sheko	Sirloin	15.13 ± 6.53 ^d^	−0.02 ± 0.02 ^e^	5.43 ± 2.76 ^cd^	2.88 ± 1.42 ^h^
Sheko	Chunck	5.68 ± 1.70 ^c^	−0.04 ± 0.03 ^e^	6.33 ± 2.28 ^cg^	2.92 ± 1.67 ^h^

Means followed by different superscripts in the same column are significantly different (*p* < 0.05).

## Data Availability

The authors confirm that the data supporting the findings of this study are available within the article.
